# Sexual Well-Being of Young People in Times of Widespread Pornography Use: Protocol for a Multidisciplinary Research Framework

**DOI:** 10.2196/80058

**Published:** 2026-05-04

**Authors:** Gunter De Win, Guido Van Hal, Michel Walrave, Joris Van Ouytsel, Koen Ponnet, Alexis Dewaele, Erick Janssen, Gily Coene, Kristien Michielsen, Sam Geuens, Inge Glazemakers

**Affiliations:** 1 SEHPO, Faculty of Medicine and Health sciences, University of Antwerp, Prinsstraat 13, Antwerp, Flanders, 2000, Belgium, 32 32654111; 2 Department of Urology, Antwerp University Hospital, Antwerp, Flanders, Belgium; 3 FAMPOP, Social Epidemiology and Health Policy, University of Antwerp, Antwerp, Flanders, Belgium; 4 Mios: Media & ICT in Organisations and Society, Faculty of Social Science, University of Antwerp, Antwerp, Flanders, Belgium; 5 Hugh Downs School Of Human Communication, Arizona State University, Tempe, AZ, United States; 6 Department of Communication Sciences, IMEC-MICT, Ghent University, Ghent, Flanders, Belgium; 7 Department of Experimental Clinical and Health Psychology, Faculty of Psychology and Educational Sciences, Ghent University, Ghent, Flanders, Belgium; 8 Department of Neuroscience, Insitute for Family and Sexuality Studies (IFSS), KU Leuven, Leuven, Flanders, Belgium; 9 RHEA Centre of Expertise on Gender, Diversity and Intersectionality, Vrije Universiteit Brussel, Brussels, Brussels, Belgium; 10 Department of Healthcare, PXL University College of Applied Arts and Sciences, Hasselt, Belgium; 11 Outpatient center for Sexology, SFZ Hospital, Heusden-Zolder, Flanders, Belgium; 12 Collaborative Antwerp Psychiatric Research Institute (CAPRI), Faculty of Medicine and Health Sciences, University of Antwerp, Antwerp, Flanders, Belgium; 13 University Center Child and Adolescent Psychiatry Antwerp (ZAS-UKJA), Antwerp, Flanders, Belgium

**Keywords:** pornography use, online pornography, sexual wellbeing, adolescents, young adults, diverse society, multidisciplinary research, sexual development

## Abstract

**Background:**

Since the rise of freely accessible pornographic streaming websites, pornography consumption has become widespread and normative worldwide. In Flanders, early exposure—before age 13—has tripled over the past decade, and frequent use, particularly among young men, is common. While pornography consumption may support body satisfaction, self-exploration, and self-esteem, evidence on its effects on sexual development and sexual well-being remains limited. Public debates are polarized, swinging between moral panic and denial of potential risks. Care providers and helplines increasingly report young people struggling with pornography-related concerns, such as self-perceived porn-induced sexual dysfunctions. Adolescents and young adults from diverse backgrounds express a clear need for guidance in navigating sexually explicit media, particularly when communication with parents, teachers, or health care providers is difficult.

**Objective:**

This project aims to generate evidence-based insights into the complex relationships between pornography consumption, sexual development, and sexual well-being among young people. By producing actionable knowledge, it seeks to inform education, prevention, and care practices that help adolescents and young adults navigate sexually explicit media in ways that promote healthy and inclusive sexual well-being within Flanders’ ethnically and sexually diverse society.

**Methods:**

The project consists of four interconnected work packages: (1) examining pornography in relation to societal norms and inequalities, (2) exploring pornography within family-based sexual development, (3) investigating pornography’s role in health care contexts, and (4) developing evidence-based pornography literacy tools for education and prevention. A mixed methods approach will combine systematic scoping reviews, a nationally representative survey, laboratory studies, qualitative interviews and focus groups, and co-creation with key societal stakeholders.

**Results:**

The project received funding from Research Foundation – Flanders in 2024, and researchers were appointed between September and November 2024. Scoping reviews began in January 2025 and concluded in October 2025. A large-scale survey will be conducted between January and March 2026, followed by subsequent stages of analysis, dissemination, and valorization, concluding in 2028.

**Conclusions:**

Although empirical results are not yet available, the project will deliver new evidence on how pornography consumption shapes sexual development and sexual well-being across diverse contexts. It will produce practical outputs for education, health care, and policy, and contribute to reducing stigma and misinformation around pornography use. By addressing pornography as a multifaceted social and sexual phenomenon, this multidisciplinary research will advance scientific understanding and promote more inclusive, evidence-based approaches to sexual health education, care, and policy.

## Introduction

### Rationale and Positioning Regarding the State-of-the-Art

#### Pornography Consumption: From Exploration to Sexual Pleasure to Decreased Sexual Well-Being

Growing access to the internet has resulted in more and earlier consumption of online pornography. Estimates of adolescent lifetime use exceed 80% [1]. In Belgium, 51.3% of boys [2-6] report having viewed pornography, and in Sweden, daily use among adolescents increased from 11% to 24% [7]. In the United Kingdom, 55% of men (aged 18‐25 y) said porn had been their main source of sex education [8]. While these experiences may be associated with positive effects on body satisfaction, self-exploration, and self-esteem [9,10], the overall impact of pornography consumption in adolescents and young adults on their general and sexual development, health, and well-being remains to be established [11,12]. The few longitudinal studies in adolescents and young adults published so far suggest that some adolescents and young adults develop problematic pornography consumption and a decreased quality of sex life in young men [13,14]. Research conducted by some of the applicants saw a higher incidence of problematic pornography consumption in those adolescents and young adults who started watching before they were 11 years of age [2]. However, a national representative study in Sweden found predominantly positive effects of pornography consumption [3]. Indeed, several studies have reported positive effects of pornography consumption (eg, increase variety of sexual activities and pleasure in long-term partners, and improve attitudes toward sexuality) [4-6,15]. On the other hand, some individuals consult health care professionals for sexual difficulties they attribute to their own pornography consumption [16], and some Flemish sexologists report increasing numbers of younger clients seeking help for pornography-related concerns [17]. Interestingly, the Swedish study referred to above reported predominantly negative effects of pornography in the age group 25‐34 years.

Some researchers have not consistently found positive or negative associations between sexual functioning and the frequency of pornography consumption [5,18]. Self-perceived “pornography addiction” has been attributed to moral incongruence toward pornography consumption, and some studies have found individual differences in, for example, affect regulation and impulse control may also play a role [19]. Whereas the *DSM-5* (*Diagnostic and Statistical Manual of Mental Disorders* [Fifth Edition]) [20] does not include a diagnosis for sexual addiction or hypersexuality, the diagnosis of compulsive sexual behavior disorder was added to the *ICD-11* (*International Classification of Diseases, 11th Revision*) in 2019. Although many potentially dysregulated sexual behaviors may result in this diagnosis, problematic pornography consumption is assumed to be one of the most encountered in clinical settings [21].

Defining pornography has been proven to be challenging, as scholars within and across disciplines have difficulty reaching a consensus on how it is best conceptualized. Most commonly, and historically, porn has been described as involving explicit representations of sexual behavior, created with the intent of inducing arousal. Other conceptualizations approach porn as a “thought structure” focusing on the individual but emphasizing cultural and temporal changes in conceptions of its content, role, and function [22]. While acknowledging that what defines pornography may differ between individuals, cultures, and time, we conceptualize pornography as “materials experienced as sexually explicit and/or arousing by the user and created with the intent to be experienced as such.” Pornography is most commonly used during and for masturbation, a generally documented positive health behavior. Furthermore, only a minority reports negative consequences from pornography consumption. However, pornography is easily accessible, and a significant percentage of adolescents and young adults (both men and women) use it frequently. It has been estimated that 17% of young men never masturbate without the use of pornography. Pornography consumption may reduce stress, induce pleasure, and increase positive mood, which is consistent with the increase in pornography consumption reported during the COVID-19 pandemic [23]. Different motivations for pornography consumption and problematic pornography consumption may be identified [24].

Given widespread pornography consumption, it may not be surprising that adolescents and young adults sometimes wonder if their use pattern is normal or problematic, if it is normal that they sometimes seem to become less responsive to it (habituation), wonder if they need to go “cold turkey” (abstinence), and parents, teachers, and others (including literacy programs and help lines) struggle to provide scientifically-based answers to these questions. There is a clear need for thorough interdisciplinary research to understand the range, extent, and predictors of positive and negative effects of pornography consumption, including possible changes in desire and sexual responsivity, but also, more generally, its associations with sexual and mental health. This project is first and foremost focused on the sexual well-being and sexual development of adolescents and young adults in the current social and sexual culture, of which porn is an important aspect. Instead of focusing on pornography itself, we will focus on (1) motivations and habits of consumption, (2) how porn fits in their (sexual) lives, and (3) how it may influence their sexual and mental health. Recent preliminary insights suggest clearly that it is not pornography consumption per se but why and how it is consumed that may best explain possibly negative or positive outcomes [25-27].

#### Pornography Consumption: Toward an Objective Interdisciplinary Approach

Throughout history, sexual values and norms have been prone to debate as they are intrinsically linked to what makes us human and how societies are structured and organized. The literature on pornography is no exception and often includes sociocultural and religious viewpoints; as such, it is polarized, resulting in several conflicting articles [28]. Previous studies have come to conflicting conclusions, especially when it comes to the question of whether pornography is related to the sexual attitudes and behavior of adolescents and young adults [29], and this may, in part, have been caused by the fact that most studies focused on pornography itself. By studying behavioral patterns and motivations of use (or nonuse), we hope, with this project, to move beyond controversies and contribute to a more balanced view. In this, we propose to put forward a framework aiming to understand the sexual development of adolescents and young adults and its relation to sexual well-being.

Our framework is consistent with a recent Delphi study, which defined 6 key competencies for healthy sexual development that have the potential to strengthen or impede adolescents and young adults’ sense of sexual well-being in relation to both themselves (eg, body image and self-efficacy) and others (eg, mutually respectful relationships). This framework further describes how adolescents and young adults’ socioecological structure influences how they can translate these competencies into actions and achieve a sense of sexual well-being. We believe that a carefully designed multidisciplinary project, where researchers from different scientific backgrounds actively work together in a standardized way, focusing on the sexual well-being of adolescents and young adults in a time of widespread pornography availability and pornography consumption (rather than pornography itself), is important to advance knowledge in this field [30]. The consortium behind this study, sexual well-being of young people in times of widespread porn use (SWYPPe), consists of a diverse group of academics (sexual education, psychology, medical sociology, pedagogy, epidemiology, sexology, psychiatry, urology, gynecology, pelvic floor, queer studies, communication and media studies, and gender diversity and intersectionality) who collaborate with societal stakeholders to further with our aim of providing an unbiased scientific analysis and to gain better insights into a (taboo) topic of little systematic academic attention [31]. This interdisciplinary research will be facilitated through the participation, collaboration, and communication with all stakeholders and between disciplines across sectors, especially in dealing with adolescents and young adults [32]. The results of this research will not only benefit individual questions for support coming from young people themselves, their parents, or their teachers, but will also inform public health interventions that often struggle to consider the lived experiences of adolescents and young adults in forming relationships and securing their health and well-being [32].

#### Informed Responsible Pornography Consumption: An Objective Definition

To define responsible pornography consumption, we first need to address the following topics:

1. An interdisciplinary perspective on pornography consumption, sexual health, and sexual well-being in our society. This knowledge is needed to inform porn consumers and health care professionals from different fields or backgrounds on what the “norm” is (most seen behavior) of pornography consumption in the diverse (both culturally and sexually) society of Flanders and its influence on sexual well-being. Internal comparison of porn consumers (and those who refrain from porn) and their motivations might help us understand why pornography consumption affects some people while not affecting others.
2. The role of pornography consumption on the sexual development of adolescents and young adults, and how parents deal with this matter. We need to address this knowledge gap to better understand the possible effects (and mediators) of online pornography consumption on sexual arousal in adolescents and young adults, and the risk or protective factors for abnormal development or behavior, and how we can facilitate parent-child communication about this.
3. Understanding of patterns and motivations of pornography consumption in relation to adolescents and young adults’ sexual well-being in health care contexts. Although only a minority experiences negative effects, the incidence of young people with sexual problems is increasing. Evaluating pornography consumption in adolescents and young adults who grew up in a time of easily accessible pornography and present with sexual difficulties will lead to a better understanding of possible risk and protective factors, inform prevention strategies, but also let us understand the possible positive impact of pornography on certain sexual problems.
4. An informed development of an interdisciplinary evidence-based porn literacy (porn literacy) lesson plan and self-assessment tools, as there is no consensus among scholars on what kind of topics these educational programs should cover and how they should be delivered [33-35]. Furthermore, it is unclear to what extent these pornography literacy programs are evidence-based, which forms of pornography they discuss, and how many have been evaluated [36].

### Scientific Project Objectives

This project is not just porn-research, as it is foremost focused on the sexual well-being of adolescents and young adults in the current social and sexual culture, where porn cannot be ignored. Our project starts from needs felt by a diverse stakeholder team with regards to sexual well-being in the current (digital) timeframe, and it also focuses on a sorely understudied population. This is largely due to funding difficulties and challenging social climates around topics, such as pornography and, more broadly, to studying sexual development in young people. Across the globe, research on pornography consumption among adolescents and young adults faces comparable ethical, cultural, and political challenges. Many countries have reported similar patterns of increasing accessibility, early exposure, and polarization in public discourse, while empirical research often lags behind social change. In this international context, Flanders offers a valuable case study—a Western European region reflecting broader societal trends in digital sexual culture, yet distinctive in its progressive research ethics framework that permits the inclusion of minors aged 14 years and older. Insights gained here are therefore not merely regionally relevant but contribute to a growing international evidence base on how young people navigate pornography within rapidly evolving media and sexual landscapes (eg, study by van Oosten et al [37]). Flanders presents us with a unique opportunity, as we are allowed to engage adolescents and young adults, starting from the age of 14 years, in research on sexual well-being, including pornography, without facing recruitment constraints that may limit the representativeness of our findings (refer to Ethical Considerations section). Previous Flemish research on sexual health has indicated that it is both feasible and important to recruit youth aged 14 and older [38]. Our SWYPPe project will expand scientific knowledge on pornography consumption and its potential effects on young people, thus creating momentum in this field of research internationally. The widespread and open availability of pornography in Belgium, together with the help of a diverse interdisciplinary research and stakeholder team, provides a unique opportunity to objectively investigate the effects of pornography consumption on several levels. The overall objective of this project is to learn how adolescents and young adults can navigate pornography in a healthy way and how measures can be developed to prevent or limit potential negative consequences from happening while still respecting the positive effects its consumption can have on the individual. The proposed research will inform us to develop applications, tools, and an awareness campaign that will help adolescents and young adults navigate pornography in an informed, responsible, and normalized way, leading to improved sexual well-being of young people [39].

### Project Management: Research Approach and Work Plan

**Figure 1.**
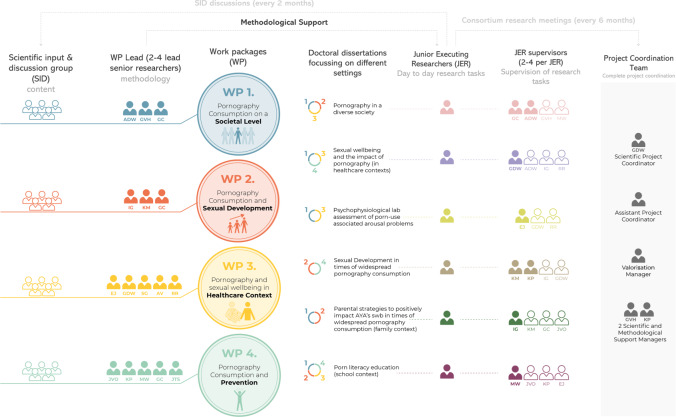
Division of project into work packages.

We define four work packages (WPs; Figure 1), focused on (1) pornography consumption on a societal level, (2) pornography consumption and its impact on sexual development, (3) pornography consumption and sexual well-being in the health care context, and (4) pornography consumption and prevention. Researchers (from different applicants) will be assigned to lead each WP. For every WP, an interdisciplinary scientific input and discussion (SID) group, consisting of experts and societal stakeholders, will be set up. Based on their field of expertise, the SID members will give input into the research questions and ensure all needs are addressed, results (preliminary) are discussed, and input for further steps will be given. Throughout the different WPs, several interdisciplinary research topics will be defined that will lead to a doctoral dissertation (PhD). These PhDs will focus on different settings (eg, societal setting, health care setting, family setting, and school setting).

## Methods

### Project WPs

#### WP1: Porn and Society—an Intersectional Perspective on Pornography Consumption, Sexual Health, and Well-Being

Research on the incidence and effects of porn has mainly focused so far on White, cisgender male populations, and on pornography consumption itself. Research also lags behind in understanding the impact of pornography consumption, among others, on sexual functioning [40] (ie, moving through the stages of sexual desire, arousal, and orgasm, as well as subjective satisfaction with the frequency and outcome of individual and partnered sexual behavior), romantic relationships, and across sexual and gender identity groups [41-43]. In this WP, we focus on the incidence, prevalence, and motivation of pornography consumption, its impact on sexual health and sexual well-being (including relationship satisfaction) [44,45], and the risk factors or positive effects of pornography consumption among different minoritized groups in society. While pornography consumption has become more accepted in Western societies, it remains controversial, and attitudes may differ to a large extent according to religious beliefs and cultural and social norms [46]. In an increasingly diversifying society, it is important to understand how porn is consumed, perceived, and interpreted within various populations, considering multiple positionalities on the basis of gender, sexuality, ethnicity, age, or class. Internal comparison of porn consumers (and those who refrain from porn) and their motivations might help us understand why pornography consumption affects some people while not affecting others [27]. This WP implements an intersectional perspective (ie, moving beyond 1-dimensional categorizations) and focuses on widening the theoretical scope and functions as a knowledge base for WP2, WP3, and WP4 (Multimedia Appendix 1).

By using a mixed methods approach, the incidence and prevalence of pornography consumption will be assessed with a specific interest in risk or protective factors, psychological well-being, and sociocultural background; complemented by a qualitative in-depth study that focuses on the impact of identities, contexts, complexities, ambiguities, and challenges.

Research objective (RO) 1.1. and 1.2.: We will start with a systematic literature review of qualitative and quantitative studies, as well as bringing together validated scales relating to psychological well-being, relationship satisfaction, and sexual dysfunction. The scoping review focuses on youth aged 14‐25 years, especially those from minoritized groups (eg, lesbian, gay, bisexual, transgender, queer or questioning, and others; ethnic minorities; youth with disabilities; different gender identities; and religion), and how they perceive and interpret online pornography. Searches were conducted in Web of Science, PubMed, Scopus, and Education Resources Information Center (ERIC) in March and August 2025. From 4160 records, 2194 duplicates were removed, and 108 full texts are currently under detailed review. Data extraction will capture participant characteristics, study design, conceptual focus, and main findings. Results will be synthesized narratively and thematically, paying special attention to intersectional perspectives.

This will be followed by a cross-sectional representative survey on a societal level. A questionnaire will be developed, taking into account the results of the systematic review and making use as much as possible of validated questions and scales (eg, studies by Cacioppo et al [47], Alidost et al [48], Rosen et al [49,50], and Grubbs et al [51]). In literature, there is substantial discussion on the measurement of sexual well-being. Research from the SWYPPe consortium has found that most well-being measures focus a lot on sexual satisfaction and sexual functioning. Sexual well-being, however, as a critical part of overall public health, includes several other domains (safety, security, respect, etc) [52]. To expand this measurement, we have done preparatory studies into the development of an adapted scale, including building an international consensus on the subconstructs of sexual well-being through a Delphi study among international researchers (n=58) and participatory research with a diverse global panel of adolescents and young adults—in terms of gender, sexual orientation, ability, and nationality [53]. This resulted in a new and inclusive framework for sexual well-being [53]. Currently, we are finalizing a validated measurable scale that will be used in the survey, by building on existing scales, such as the Short Sexual Well-Being Scale, and integrating aspects of the new framework [54]. By setting up a large-scale representative survey in Flanders, we will gather extensive information on pornography consumption and sexual health on the one hand, and demographic, psychological, relational, and sociocultural correlates on the other hand [38]. For this survey, participants aged between 14 and 25 years will be selected by using a sample frame of 15,000 from the Belgian National Register (via Statbel) to end up with a net sample of at least 3000 respondents. A sample size of 3000 also allows for subgroup analyses. For any subgroup analyses, we will correct for multiple testing. For the sake of multiple testing, in subgroup analyses with 500 respondents, we will work with a Cronbach α of 0.01, still having good power to find an effect size of 0.20 (power=0.97). Based on a power analysis (using G*Power, *t* test correlation), with an effect size of 0.20 (ie, a small effect), α=.05, the achieved statistical power is approximately 0.80 for a sample size of 191. Even when conducting multiple and multivariate analyses, the power to detect significant associations should still be sufficiently high (refer to a previous Flemish study [38]). An online survey will be developed. After 3 weeks (and 2 reminders), nonrespondents will be replaced by matched substitutes to get as close as possible to representativeness. Given the topic sensitivity, we will assess sexual porn-related experiences through a highly anonymized representative survey, while data on opinions, attitudes, and motivation toward porn-use (that have a less threatening nature) will further be gathered through focus group discussions and in-depth interviews (IDIs). This will help us understand to what extent (problematic) pornography consumption is associated with contextual or psychological factors and how individuals experience pornography consumption in their daily lives. Also, the positive effects of pornography consumption will be assessed, as well as its relation to psychological well-being and relationship functioning.

RO 1.3. and 1.4.: Conducting a meta-ethnography assessing the current available literature will set the scene for further qualitative inquiry into the readings of pornographic materials in various minority populations. Given the sensitivity of the topic, qualitative data on personal experiences will be collected through semistructured interviews (aligned with WP2) and through focus group discussions. We will purposefully sample (via snowball sampling and via key stakeholders such as Wel Jong, Sensoa Bazz, and Blue Ocean) a diverse mixture of youth between 14 and 25 years old that are stratified into 3 age groups (14‐15 y, 16‐18 y, and older than 18 y). Furthermore, 6 focus groups (2 for each age group) will each count an average of 8-12 participants. In total, 24 IDIs will be audio-recorded and transcribed verbatim for further analysis. The proposed sample sizes reflect the consideration of a diversity of experiences, feasibility, and comparable previous research [55,56]. All participants are thoroughly informed, provide informed consent, and their privacy will be protected by strictly following General Data Protection Regulation regulations. Besides educational background or track (in case of minors), we will monitor maximum variation in terms of spoken languages at home, socioeconomic status, gender, gender identities, and sexual orientation. Data will be analyzed using NVivo 11 (Lumivero) and through thematic analysis [57]. To grasp the unique experiences of multipositioned individuals through an intersectional lens and avoid 1-sided interpretations and constructions of relevant themes, the coding team will be diversely composed and include researchers with different demographic backgrounds.

SID group: Researchers from University of Antwerp (UAntwerpen), Ghent University (UGent), VUB Brussels University, Antwerp University Hospital, Hogeschool PXL (PXL), and Katholieke Universiteit Leuven (KULeuven), supported by Bazz, Sensoa, Vereniging van Vlaamse Seksuologen (VVS), Unesco chair sexual health, and international experts.

#### WP2: How Pornography Consumption Impacts Sexual Development and How Parents Deal With This Matter

WP2 aims to study the effects of online pornography consumption on the sexual development (“progression toward sexual maturity, in attitudes and behavior as well as in physical characteristics” [58]) and sexual well-being. Further, this WP focuses on the family context as parenting and open parent-child communication may act as a protective factor for possible negative outcomes of pornography consumption on adolescents and young adults [59,60].

RO 2.1.: The first step is to identify the available evidence and best practices. This will be done in 2 phases. First, a scoping review of scientific and gray literature. The review will examine the extent (ie, size), range (variety), and nature (characteristics) of the evidence on online pornography consumption and sexual development and sexual well-being of adolescents and young adults. We will use a predefined search syntax in scientific databases, with researchers selecting articles and data extraction. They will interact with the literature review of WPs 1 and 4. Furthermore, we will conduct an online search to identify best practices in this field. Second, expert interviews—to complement the scoping review, we will do IDIs with 10 national and international experts in youth sexual development and sexual well-being. We will identify the experts in collaboration with the consortium, as well as through phase 1. Interviews will be transcribed, and grounded coding will be applied.

RO 2.2.: To examine the effects of pornography consumption on sexual development and sexual well-being of adolescents and young adults, we use young people themselves as the main data source. We will use two data collection methods: (1) a qualitative content analysis of helpline data and (2) IDIs with adolescents and young adults (aligned with WP1). First, in qualitative content analysis, an initial analysis of the AWEL chat (ie, an online helpline for adolescents and young adults) indicated that the word “porn” gave 219 hits in 2019 and 284 in 2020. In these chat messages, young people indicate what they are concerned about regarding porn. We will do an extensive text analysis of multiple years (2018‐2023) of the AWEL chats. This will allow us to (1) identify key problems and concerns that adolescents and young adults report and (2) identify potential obstacles for youngsters to talk with their parents. Second, we will use IDIs with a focus on young people (14‐24 y) to study the self-reported perceived effect that online pornography consumption has on their sexual development and sexual well-being, including their self-image, perception of sexuality and relationships, and their preferred sexual script. By doing so, we can map their support needs and identify possible solutions. The older group (18‐24 y) has more experience and can reflect in retrospect about the process they went through, while the younger group (14‐17 y) will be able to reflect on ongoing experiences and changes. We will cluster the adolescents and young adults in different groups and aim to include at least 5 young people from each group for both age categories: (1) frequent and occasional users, (2) boys and girls, (3) heterosexual and sexual minorities, and (4) dominant ethnicity and ethnic minorities. Respondents will be recruited through open calls on social media, through the help of stakeholder Pimento targeted blogs and websites, and through health care professionals and youth welfare workers. Furthermore, respondent-driven sampling will be used. We will work with the 24 respondents from WP1 (adding additional topics to the WP1 interview guide) and complement these to reach approximately 40 interviews (until data saturation and information power is reached [61]). Pseudonymization will be performed. The interviews will be built around a timeline exercise that allows the respondents to recall the start of pornography consumption and the subsequent evolution of the course of the years. The interviews will also focus on the support needs of adolescents and young adults, in particular within the family.

RO 2.3.: To examine the needs of parents in dealing with pornography consumption of their children, the following methods will be used:

1. Online survey with parents: In close collaboration with WP1, a purposeful sample of 500 Flemish parents will participate in an online survey, constructed around both closed-ended and open-ended questions. Topics in the survey would be mediation strategies from parents on their children’s online screen time, their own experiences of pornography consumption, but also relationship quality and communication strategies between parent and their child.
2. IDIs with parents: The IDIs will focus on how parents deal with their child’s online porn viewing (intentional or unintentional), as well as on their support needs. How do they view their role in sexuality education? Do parents have other needs in supporting their children in dealing with their child’s online pornography consumption? In total, 20 parents will be recruited with special attention to the recruitment of parents coming from a vulnerable background (eg, parents from different cultural backgrounds, single parents, and lower socioeconomic status).

RO 2.4.: To inform the development of a tool to enhance communication between parents and their children, based on the results of RO2.1, RO2.2, and RO2.3, two steps will be taken:

1. Cocreation: Using the method of cocreation with parents and young people, together with the expertise of the valorization partners, we will develop an intrafamily communication tool on pornography. Parents, adolescents, and young adults from RO2.2 and RO2.3, together with the youngsters of our sounding board, will be invited to this process.
2. A proof-of-concept study: Does the tool fit the needs of children (RO2.2) and parents (RO2.3)? Does it help them communicate with each other on the topic of online pornography consumption? We will use a realist evaluation, looking beyond individual factors by taking into account context, mechanism, and outcome [62]. We will use an assessment in 15 parent-child dyads who used the tool, focusing on the presence or absence of certain communication behaviors in parents or children (identified in RO2.1, RO2.2, and RO2.3) and the outcomes they perceive. The result is an adapted tool.

SID group: Researchers from UAntwerpen, KULeuven, representatives from Agentschap Opgroeien, Sensoa, Pimento, Bazz, and external (international) experts.

#### WP3: Toward a Better Understanding of Pornography Consumption in Relation to Adolescents and Young Adults’ Sexual Well-Being in Health Care Contexts

Unraveling the interplay of pornography consumption, masturbation, and sexual habits, personality, moral viewpoints, and well-being in people who grow or grew up in a time of easily accessible pornography and later present with sexual difficulties, will lead to a better understanding of possible risk and protective factors, inform prevention strategies, and understand the possible positive impact of pornography on certain sexual problems. When speaking of pornography consumption, we are referring to adolescents’ and young adults’ patterns of porn use and motivations. This includes pornography consumption with or without masturbation, frequency, habituation effects, experience of tolerance, motivations to use (eg, to forget negative emotions or enhance arousal), changes in frequency of use (including abstinence), type of porn used, etc.

RO 3.1.: Adolescents and young adults with a diverse range of sexual problems will be included in a questionnaire-based study. Their health care worker (a general practicioner, members of professional associations such as Belgische Vereniging voor Seksuologie [BVU], Vlaamse Vereniging voor Seksuologie [VVS], Birth Core and Pelvic Therapy [BICAP], and/or Vlaamse Vereniging voor Obstetrie en Gynaecologie [VVOG]) will define the type of sexual problem and present them with an anonymous REDCap (Research Electronic Data Capture) questionnaire based on the systematic review (WP1), our preparatory survey [2], and our extensive clinical experience. Apart from our sexual well-being framework and validated scales (problematic pornography consumption) [47,48,58,63-66], the prevalence, extent, and motivations of pornography consumption in both young people and their eventual partner, their sexual experimentation, and risk-taking behavior will be questioned. Based on this study, we will explore the epidemiology of pornography consumption in adolescents and young adults with different sexual dysfunctions and compare this with the societal data (WP1). Descriptive statistics will be used to understand for which sexual problems pornography consumption needs to be studied further. To acquire generalizability of data, we will motivate clinicians from the different Flemish organizations (see above) to recruit.

More information on the process: Patients who present at various health care providers (ie, psychologists, sexologists, physiotherapists, psychiatrists, urologists, and gynecologists) for a sexual dysfunction (ie, erectile dysfunction, inability to orgasm, arousal deficiencies, and lubrication difficulties) will be asked to fill in a questionnaire to explore the epidemiology of pornography use in this population. This questionnaire will touch upon:

- Background information
- Prior sexual experiences: both masturbation and partnered sex
- The nature of their experienced sexual dysfunctions: self-report and a few validated scales.
- Psychological comorbidities: presence of attention-deficit/hyperactivity disorder, obsessive-compulsive disorder, autism spectrum disorder, depression, anxiety, and diagnosis and medication.
- Pornography use: self-report, habits, and a few validated scales on problematic pornography use, such as Cyber Pornography Addiction Test [47] and Problematic Pornography Consumption Scale [48].

Participants: All patients who present at the participating health care providers who fulfill the inclusion criteria will be asked whether they want to fill in the survey. The inclusion criteria are the following:

- Being male or female
- Speaking Dutch or English
- Presenting at a Flemish health care provider
- Self-reporting some sort of sexual dysfunction, different from “pornography addiction” or “problematic pornography use.”
- Being between 16 and 35 years old.

RO 3.2.: A phenomenographical study was based on clinical and sexological assessment and follow-up. As porn is meant to arouse, here we will specifically focus on young patients (<40 y) with sexual arousal or orgasm dysfunctions that will be recruited via clinicians (MDs and sexologists). The questionnaire on the prevalence of pornography consumption linked to research objective 3.1 will be presented. To men (and their possible partner), we will present a pictographic quiz linked to tactile sensations (Erection Hardness Score) to assess their idea of erection normality [59]. Clinical urological evaluation will assess normal development. Nocturnal erections will be evaluated, and if necessary (to rule out physical problems in men), penile duplex-doppler will be performed. In women, if deemed necessary, a genito-pelvic examination and assessment of the pelvic floor will be performed. Next, sexological case-by-case interviews with a protocol based on the sexual response cycle and sexual excitation and inhibition scales will help us identify key elements in diagnosing the arousal or orgasm problems and differentiate from performance anxiety, relational factors, distractions, habituation, need for novelty, and personal or moral views on porn as well as self-perceived addiction. Also, the extent of pornography consumption (current or previous in case of sudden abstinence), as well as the subjective experiences and motivations for pornography consumption, will be assessed. Are adequate levels of both physical and subjective arousal still attainable for this subset of patients when engaging in real-life sex with their partner of choice? A grounded theory approach will be used with inductive iterative analysis of the data captured and subsequent matrix queries with Nvivo 1.7.1 (Lumivero) as qualitative software. Interpretative data analysis could provide a more detailed insight into pornography consumption habits and the self-perceived effects of pornography consumption (recorded as closely to the subjective patient experience as possible, with the help of clinical diaries). Both data analysis of the body of cases and a focus group study with the clinical sexologists delivering the interviews will be necessary to generate detailed insights and allow to identify different subcategories of patients with arousal and orgasm problems in relation to their current pornography consumption, the motivation of their pornography consumption, and their views about pornography consumption. Iterative cycle sampling will be used to reach data saturation based on the proposed categories. These will then be tested by participating clinicians while seeing patients consulting with arousal problems during the last project year.

For a randomized controlled trial (RCT) about abstinence, we will recruit male and female participants with daily pornography consumption and problematic pornography consumption for a 2-month pornography abstinence experiment and weekly evaluation of arousal, craving, anhedonia, masturbation, sexual activity, and sexual dysfunction. Participants will be randomly assigned to an abstinence or nonabstinence group. With a Cohen *d* of 0.5, α=.05, power=0.8, and df=2, a sample of 164 participants will be needed. Based on our previous work, prevalence, and participants’ comments, we are convinced this target can be reached.

More information on the process of RCT: adolescents and young adults who use pornography will be randomly divided into a control and treatment group, during which the treatment group will abstain from pornography for 3 months to investigate the impact of pornography use on their sexual functioning and the effect of sudden abstinence from pornography on this sexual functioning. During this 3-month period, participants will be frequently asked to fill in a diary survey to assess their sexual functioning, satisfaction, and arousal.

Participants: The aim is to include 164 participants (estimated amount with Cohen *d*=0.5, α=.05, power=0.8, and df=2). All participants should fulfill the following inclusion criteria:

- Being cisgender males
- Living in Flanders
- Being between 16 and 35 years of age
- Being a frequent pornography user, defined as watching pornography in general 8 or more times out of 10 masturbation sessions
- Being a more problematic pornography user, defined as scoring above 23 on the Cyber Pornography Addiction Test Scale.

RO 3.3. Sexual psychophysiological testing: to allow for the selection of stimulus conditions that contribute the most to clinical decision making (in terms of diagnosis and treatment selection), we will compare men with erectile problems who do (group 1, n=40) or do not (group 2, n=40) report high pornography consumption, and age-matched control groups of men without erection problems who report high or low pornography consumption (group 3, n=40 and group 4, n=40) using genital response and self-report measures of sexual arousal. More specifically, we will test their (1) sexual responsivity (by presenting self- and researcher-selected erotic videos), (2) habituation proneness (by repeating the same 1-min erotic film 10 times), (3) need for novelty (through the inclusion of a compilation video of short and varied erotic video clips), (4) sensitivity to distraction (by combining an erotic film with an arithmetic task), and (5) to performance demand (induced by combining an erotic film with the instruction to become aroused as quickly as possible). Participants will also be asked to achieve their strongest erection by viewing and scrolling through their videos of choice. The laboratory assessment will be complemented with a home-based daily diary study to examine, in the same sample, similar processes (self-reported erection levels, impact of distraction, performance demand, etc) during solitary pornography use and sexual activity with a partner. With an effect size ƒ of 0.25 (medium effect), α of .05, power of 0.90, and 4 groups with 6 within-subject conditions, we need a sample of 140 participants [67]. For between or within-subject interaction effects, our proposed sample of 160 participants will have sufficient power to detect small to medium effects. Although there are fewer reports of and studies on porn-associated sexual dysfunctions in women, and although sexual response measurement in women is constrained by the relative nature of currently available measures, a pilot study will be conducted to evaluate the feasibility of this approach to the evaluation of sexual problems in women (n=40).

RO 3.4. Health care practitioner (HCP) opinions: focus group discussion (with a diverse group of HCPs, often confronted with patients with sexual problems) will be used to gain insights into the normative views that diverse HCPs hold on the effect of pornography consumption on sexual functioning and sexual well-being, and the possible link between their practice and views. Results will be strengthened by IDIs with health care workers and their patients. Once HCPs use our tools, we will evaluate and reassess their opinions.

SID group: researchers from PXL, UAntwerpen, KULeuven, Antwerp University Hospital, UGent, representatives from Sensoa, VVS, BVU, VVOG, European Sexual Medicine Network, and international experts.

#### WP4: Self-Assessment of Pornography Consumption and Porn Literacy Education

To develop inclusive pornography literacy and self-assessment resources that can help individuals of diverse backgrounds to educate themselves about the benefits and risks of pornography and to reach out for professional help, a systematic review was conducted.

RO 4.1. Systematic review: We will conduct a systematic review of porn literacy programs using similar methodologies as studies on advertising or media literacy programs [68,69]. We will conduct a systematic search of porn literacy education programs to (1) generate an overview of available porn literacy resources, (2) analyze their contents, (3) assess their didactical and pedagogical quality, (4) evaluate their shortcomings, and (5) identify critical areas for future development of porn literacy programs.

The systematic review will follow a scoping review methodology, applying the Joanna Briggs Institute framework [70] and reporting in accordance with the PRISMA-ScR (Preferred Reporting Items for Systematic Reviews and Meta-Analyses extension for Scoping Reviews) [71] checklist. The review will be structured using the population-concept-context framework [61].

- Population: Empirical studies involving adolescents and young adults aged 12‐25 years, including studies where the mean age falls within this range.
- Concept: Positive effects of pornography use, categorized into 6 predefined domains (knowledge, emotions or affect, norms or attitudes, sexual behavior, sexual identity or self-image, and relationships or interpersonal communication), supplemented by sexual functioning and other benefits.
- Context: All geographical regions will be considered, acknowledging the global accessibility of pornography.

Search strategy: Systematic searches will be conducted in Web of Science, PubMed, Scopus, and ERIC (April 2025), using a combination of Medical Subject Headings terms and free-text keywords. The strategy will be refined iteratively to optimize both relevance and comprehensiveness.

Study selection: The following steps were used for study selection:

- Duplicates will be removed using EndNote (Clarivate) and Covidence (Veritas Health Innovation),
- Title and abstract screening will be performed independently by 2 reviewers,
- Full-text screening will be conducted by the lead reviewer, and
- Inclusion criteria will focus on age, study type (peer-reviewed empirical research), language (English), and reporting of positive effects.

Data extraction and analysis: Data will be extracted using a structured form in Covidence, piloted and verified for completeness. Positive effects will be categorized using the sexual media practice model [62]. Contributing factors will be interpreted through the differential susceptibility to media effects model [72]. Analysis will follow a descriptive approach, including frequency counts and narrative synthesis.

Participants and recruitment: As this is a scoping review, no new participants will be recruited. The review will include studies involving adolescents and young adults, conducted across diverse countries and contexts.

RO 4.2. Development and validation of a new pornography literacy scale: Based on the review (RO 4.1.) and preliminary data that were collected in the preproject phase, as well as discussions with stakeholders, we will construct a Pornography Literacy Scale, which aims to measure the understanding and critical engagement with pornography. Both qualitative and quantitative content validity will be examined; an expert panel of scientists and practitioners (eg, Sensoa, part of SID, and WP4) will evaluate the content, wording, and scaling of the scale, and the content validity index and the content validity ratio will be calculated. Each item’s clarity, simplicity, and relevance will be measured by the content validity index [73]. The content validity ratio tests the item-essentiality [74]. The scale will be tested using a cross-sectional study with 200 respondents who are diverse in gender, sexual orientation, and cultural backgrounds. A sample of 5 to 10 individuals per item is required to ensure a theoretically clear factor structure for exploratory factor analysis and confirmatory factor analysis [75]. The sample size was determined to be 200 adolescents and young adults for each phase of construct validity (maximum 20 items×10 participants) [2,76]. We will adapt this sample size to the number of included items. We will carry out exploratory and confirmatory factor analyses and test the convergent or discriminant validity and the internal consistency of the scale (using Cronbach α and composite reliability).

RO 4.3.: To assess the perceived needs of adolescents, young adults, and teachers with regard to porn literacy education, we will, first, conduct a cross-sectional survey among a diverse sample of 2500 middle- and high-school students between 14 and 18 years old in Flanders who are diverse in gender, sexual orientation, and cultural background, reflective of the diversity of the school population in Flanders. While the representative survey in WP1 focuses on the prevalence and correlates of pornography use among the broader population, the survey in this WP4 specifically hones in on the variables relevant to creating a porn literacy module. Additionally, it aims to evaluate the role of porn literacy in mitigating the impacts of pornography consumption among young people. The questionnaire will measure (1) sociodemographics; (2) different types of pornography consumption, pornography-related attitudes, subjective norms (ie, adolescents and young adults’ perceived norms concerning pornography consumption), pornography-related behavioral intentions [77], problematic pornography consumption (also refer to WP1); (3) porn literacy, using our own developed pornography literacy scale; and (4) expectations regarding porn literacy education, based on focus group studies (eg, study by Dawson et al [35]), we present students with topics or themes and pedagogical modalities of porn literacy education that were identified by focus groups as important aspects. Students will be asked to evaluate the topics and themes, and how these would ideally be delivered (also open-ended questions will ask which topics they want to see addressed). We will also include (5) mental health variables and (6) sexual (risk) behaviors, both online and offline. We have extensive experience with conducting surveys on sensitive issues with diverse young people’s populations, and we will use the established IRB protocols that we have successfully used in previous projects on youth sexual behavior to secure participant informed consent. Quantitative analyses could include:

- Descriptive statistics to map prevalence and patterns.
- Regression analyses to examine associations between porn literacy and pornography consumption–related outcomes.
- Moderation or mediation models to assess the role of porn literacy in mitigating the impact of problematic pornography consumption.
- Subgroup analyses by gender, sexual orientation, and cultural background.

Participants will be recruited through collaboration with Flemish secondary schools, selected to ensure variation in school type, region, and socioeconomic background. Schools will be contacted via existing networks, educational umbrella organizations, and direct outreach. Parental consent and student assent will be obtained in line with ethical guidelines for research with minors. The study builds on previous experience conducting sensitive surveys with adolescent populations and will follow established IRB protocols previously approved for youth sexuality research.

Second, conduct IDIs with teachers: we will conduct IDIs (n=30) about porn literacy education with secondary school teachers responsible for relational and sexual education. The teachers will be recruited from various schools across Flanders and will be balanced in their socioeconomic status. We will evaluate what, in their opinion, can be a feasible way to deliver digital tools and porn literacy lesson plans, and which challenges they face regarding porn literacy. Interview data will focus on:

- Teachers’ perceptions of porn literacy and its relevance in relationship and sexuality education.
- Barriers and facilitators to implementation.
- Suggestions for pedagogical approaches and curriculum integration.

Interviews will be transcribed and analyzed using thematic analysis, following the approach by Braun and Clarke [78]. Coding will be both inductive and deductive, guided by previous literature on sexuality education and digital pedagogy. NVivo or similar software will be used to support coding and theme development. Teachers will be recruited via school networks, professional associations, and direct outreach to schools. Selection will aim for diversity in teaching experience, school context, etc. Participants will be invited to share their perspectives voluntarily and will provide informed consent.

RO 4.4. Evidence-based lesson plans on porn literacy: we will develop an educational program on porn literacy that is tailored toward the needs of Flemish adolescents and young adults. Our review revealed that existing programs are predominantly Anglocentric in their focus [34,77,79]. While some programs were mindful of ethnic and cultural diversity in pornography consumption and effects, they were often not cocreated with a diverse group of youngsters and experts. They were not designed to address the unique needs of culturally and ethnically diverse youth in Flanders and seemed to be centered around a negative framing of pornography, whereas our program aims to equip adolescents and young adults with a nuanced understanding of its potential benefits and risks. Our lesson plans’ general aim will be to increase young people’s knowledge and critical attitudes toward pornography consumption (including the various business models of pornography, how it can empower those in the industry), and to teach them how to assess and self-monitor their pornography consumption, to identify the potential benefits of pornography consumption, to be aware of various cultural approaches to pornography consumption, and provide them with resources in case they need professional help regarding their pornography consumption. The plans will specifically address the needs of adolescents and young adults in Flanders and educators who were identified throughout the various stages of WP4 and the other WPs. A specific outline of the topics will be based on the results of the questionnaire among youngsters (WP1 and 2) and parents (WP2). A senior researcher with extensive experience in lesson plan development will make sure that the lesson plans add to students’ learning in this context and that they are innovative, age-appropriate, and pedagogically sound. We will regularly collect recommendations (workshops with stakeholder-experts who closely collaborate with schools (eg, Sensoa and Pimento) and various teachers (including schools with students from a diverse cultural background). The design will be theory-guided, for example, Prototype Willingness Model [80], Health Belief Model [67], or the Stages of Change Model (transtheoretical model) [81]. We will use and test a variety of pedagogical approaches and make sure that the program uses inclusive language and is sex-positive. We will be mindful of the needs of adolescents and young adults (including lesbian, gay, bisexual, transgender, queer or questioning, intersex, and asexual, aromantic, or agender diverse cultural backgrounds). We will organize cocreation workshops with young people, teachers, and experts, building on our long-standing expertise. Pre- and posttest assessments of the lesson plans (including control groups) will be performed (around 600 students, including 300 in the control group). Pornography-related attitudes, behavioral intentions, and levels of porn literacy, etc are expected to be positively affected by our lessons.

RO 4.5. Development of a self-assessment tool: based on the results of the previous WPs, a self-assessment tool for porn consumers will be developed (including expectations and ideas about sexuality). Based on our risk analysis, the influence of pornography consumption on their sexual functioning will be analyzed. If needed, trained clinicians are shown.

SID group: researchers from UGent, UAntwerpen, PXL, VUB Brussels University, and representatives from Sensoa, Pimento, AWEL, EXPOO, and the UNESCO chair of Sexual Health.

### Reporting Standards and Scope of the SWYPPe Project

This manuscript presents a high-level umbrella protocol describing the overarching methodological framework and planned research approaches of the SWYPPe project, a multiyear, multidisciplinary research program consisting of multiple interconnected WPs, spanning 6 interconnected PhD projects, each of them containing multiple different substudies, with their own specific methodological setup and design.

While this protocol outlines the intended study designs, populations, and analytical strategies across the 4 WPs and 6 PhD projects, detailed study-level protocols (eg, for individual scoping reviews, experimental studies, RCTs, and experience sampling method studies) will be developed, submitted for ethical approval, and reported separately before data collection and analysis.

Consequently, all study protocols of each separate substudy within the SWYPPe project will be operationalized in line with internationally recognized reporting standards to ensure methodological transparency and reproducibility. Specifically, they will draw on the SPIRIT (Standard Protocol Items: Recommendations for Interventional Trials) 2013 Statement [82] and its relevant extensions for protocol reporting, the STROBE (Strengthening the Reporting of Observational Studies in Epidemiology) [83] guidelines for the quantitative cross-sectional components, the PRISMA-P (Preferred Reporting Items for Systematic Reviews and Meta-Analysis Protocols) guidelines for review studies, and the COREQ (Consolidated Criteria for Reporting Qualitative Research) checklist [84] or the qualitative interview and focus group components. Together, these frameworks will inform the structure, level of detail, and ethical considerations presented in this manuscript.

Some of the guidelines cannot be reported at this stage in the SWYPPe project, as not all substudies have yet started. For others, like the PRISMA-P and SPIRIT guidelines, they are within this manuscript, applied at the level of planned methodological approach rather than at the level of finalized study protocols. Items that require study-specific operationalization (eg, finalized eligibility criteria, search strategies, randomization procedures, or outcome definitions) are therefore indicated as not yet applicable at this stage and will be fully addressed in subsequent study-level protocol publications. Attempting to include the necessary level of detail needed, typically, for example, a PRISMA-P checklist for all 3 scoping reviews to be conducted within the SWYPPe project would not be feasible nor desirable in this manuscript.

### Ethical Considerations

At the present stage, only systematic reviews and 1 secondary analysis of previously collected clinical data are actively being conducted. For this latter component, ethical approval was obtained at the ethics board of the University Hospital of Antwerp (Project ID 7832 - EDGE 004438). Before the initiation of primary data collection, the complete SWYPPe project will be submitted for ethical approval to the Ethics Committee of the Faculty of Humanities at the University of Antwerp, which serves as the coordinating institution. When required, additional study-specific approvals will be obtained for WPs that involve clinical contexts or direct interaction with participants (eg, WP3), building upon the general approval. Informed consent will be obtained for all relevant studies after the nature and possible consequences of the research are explained. All research activities will adhere to the principles outlined in the Declaration of Helsinki and applicable Belgian and European data protection and research ethics regulations.

### Data Management

At this stage of the SWYPPe project, no new datasets have yet been generated, as the studies currently underway consist of systematic reviews and 1 secondary analysis of previously collected and anonymized clinical data. All future data collection will follow the project’s approved data management plan, which was developed in accordance with FAIR (Findable, Accessible, Interoperable, and Reusable) principles and the open science policy of the Research Foundation – Flanders.

Because SWYPPe brings together multiple universities, research teams, and WPs, the data management plan is necessarily complex. Anticipating the need for clear data governance and availability, these arrangements have been formalized in the consortium’s partnership agreement. The agreement stipulates that data will be stored according to each partner university’s institutional policies and infrastructure, while also allowing for controlled data sharing with third parties for research purposes. Given the societal sensitivity of the topic, any external data access requests must be communicated to the project’s principal investigators and evaluated within the predefined ethical and governance framework.

Upon project completion, anonymized datasets and accompanying documentation will be deposited in an institutional repository hosted by the University of Antwerp. Access to these data will be granted to qualified researchers following approval of a motivated request that aligns with SWYPPe’s ethical principles and its commitment to neutrality and interdisciplinary integrity.

## Results

### Overview

The SWYPPe project is structured as a multiyear research program (2025‐2028) consisting of 4 interrelated WPs. Results will be generated in a phased manner, with iterative integration across WPs.

### 2025-Early 2026

During this initial phase, the project will generate foundational evidence and prepare large-scale empirical studies.

- Scoping reviews addressing (1) pornography use among diverse youth populations, (2) parent-child communication about pornography, and (3) positive aspects of pornography use will be completed and submitted for publication by summer 2026.
- Development of core survey instruments will be finalized, informed by expert and stakeholder feedback.
- A pilot study for the national population survey will be conducted in January 2026.
- A Daily Diary study will be ready for data collection. Literature review, development of materials, and research design will be completed. Ethics approval will be applied for, and piloting of the study will have begun.

### 2026

This phase will focus on large-scale data collection across population, family, health care, and educational contexts.

- The national representative survey on pornography use and sexual well-being will be launched in April 2026.
- Parent-child dyadic survey data collection will commence, focusing on family communication around sexual topics.
- Survey data will be collected on pornography use and the sexual development and well-being of adolescents and young adults.
- Clinical survey data collection will begin among health care populations.
- Development and expert validation of the pornography literacy scale will be completed, followed by pilot testing.
- Qualitative expert interviews related to health care, sexual well-being, and development of adolescents and young adults, and educational contexts, will be conducted throughout the year.
- Data collection for Daily Diary 1A has begun in early March 2026 and will span around 6 months, given the 30-day study period.
- Literature review and design of the psychophysiological laboratory study will start (unless priority is given to Daily Diary 1B). This study is estimated to be highly time- and labor-intensive and will continue into 2027.
- Preparations for Daily Diary 1B will begin soon after the initial results of Daily Diary 1A have been obtained.

### 2027

In 2027, the project will shift toward integration, intervention development, and experimental testing.

- Analysis of survey data across WPs will be completed and disseminated through academic publications.
- An RCT examining pornography abstinence in a health care context will be completed by mid-2027.
- In-depth qualitative interviews with adolescents, young adults, and parents will be finalized.
- Development and implementation of a pornography literacy education program will take place, informed by survey and qualitative findings.
- Family-focused and educational communication tools will be developed based on integrated evidence.
- Data collection will begin for Daily Diary 1B (clinical sample).
- The design and materials of the psychophysiological laboratory study will be finalized. Data collection will commence and is estimated to last all year and carry into 2028.

### 2028

The final phase focuses on evaluation and knowledge translation.

- Proof-of-concept and realist evaluations of developed tools (family communication tools and pornography literacy interventions) will be conducted.
- A phenomenographical qualitative study examining lived experiences related to pornography use and sexual well-being will be completed.
- A self-assessment tool for young people, contingent on successful previous phases, will be developed and refined.
- Final publications and synthesis outputs will be completed, providing evidence-based recommendations for health care, education, families, and policy.
- Two small pilot studies concerning (1) daily diary and (2) psychophysiological laboratory study will be conducted with a sample of young adult women. Data collection for all studies will be finalized. Synthesis of outputs and manuscript will start and will be finalized in the first quarter of 2029.

**Figure 2.**
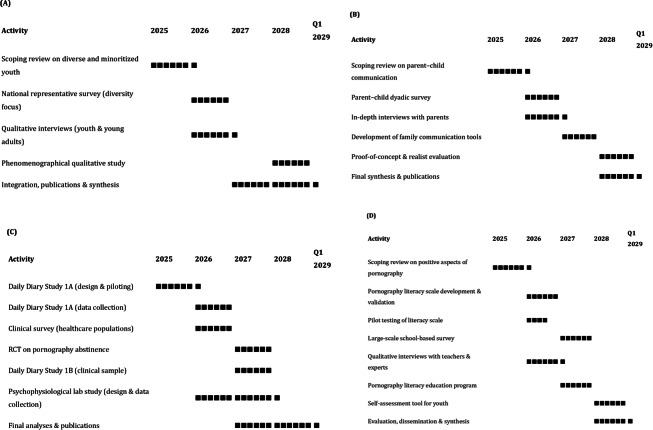
Timeline of project. (A) WP1 – Diversity, pornography use, and sexual well-being; (B) WP2 – Sexual development and family context; (C) WP3 – Health care and sexual well-being; and (D) WP4 – Educational context and pornography literacy.

An overall Gantt chart describing the timeline for the project is provided in Figure 2.

## Discussion

### Principal Findings

While many existing studies on adolescents and young adults, and pornography consumption have been framed through preexisting moral, cultural, or ideological assumptions—often resulting in hypotheses that position pornography as either inherently harmful or inherently beneficial—our project deliberately takes a different approach. The SWYPPe study does not start from predefined expectations about the direction or nature of outcomes but aims to provide an evidence-based and balanced understanding of how pornography fits within the broader sexual well-being of young people. This open, exploratory stance allows us to move beyond polarized debates and to generate findings that reflect the lived realities of youth rather than the assumptions of researchers or funding bodies. Consequently, the present protocol emphasizes methodological rigor, inclusivity, and interdisciplinarity rather than specific hypothesized results.

In the following section, we therefore outline potential methodological and organizational risks across the different WPs, along with corresponding contingency plans, to ensure that this exploratory and interdisciplinary design can be implemented robustly and ethically.

### Risk Analysis and Contingency Plans

#### WP1

One of the biggest threats could be that the response on the questionnaire, IDIs, and focus group discussions is (too) low, and that minority groups are not well-represented. This is particularly a threat for the survey that aims at a sample of 3000 participants. As explained in the power analysis, with a power of 0.8 for smaller subgroup analysis, 191 participants (6.3% of the respondents who identify in that subgroup) are required. If only 4% to 6% of the population identifies within a certain subgroup, our sample would contain between 120 and 180 participants. This will not allow differentiation (much) within this population. However, we will still be able to compare minority groups with majority groups in terms of key variables. Also, the qualitative studies focus on maximum variation. In these studies, representativity is not the aim, yet including “voices” from individuals with diverse characteristics (eg, in terms of gender identity and ethnicity) is. The quantitative survey will allow us to compare between groups, while the qualitative studies allow us to view processes and experiences in pornography consumption from a cross-sectional perspective. Our team is experienced in conducting community-based cross-sectional and participatory research. Moreover, thanks to the broad spectrum of social partners, which will strongly support the recruitment, the threat of limited representation will be limited. If necessary, an independent recruitment bureau will be engaged. The researchers are also experienced in countering socially desirable answers.

#### WP2

The main risk in this WP lies with the recruitment of participants. Given the topic sensitivity, there is a reasonable chance that recruitment of adolescents, young adults, and parents will be difficult. We will use a diversity of recruitment methods, including respondent-driven sampling, which has proven to be successful in hard-to-reach populations. In case that not sufficient adolescents, young adults, and parents can be recruited, our contingency strategy is threefold: (1) expand third-party observations in the form of additional expert interviews, (2) set up online data collection methods in the form of anonymous interviews, and (3) set up a short online survey specifically targeting adolescents and young adults for additional data collection. Our diverse group of stakeholders will also assist us in the recruitment process.

#### WP3

One particular risk may be participant enrollment. Possibly, an insufficient number of patients are willing to participate in an assessment of their pornography consumption. Therefore, all clinical stakeholders will help recruit patients. However, our earlier studies indicated that several patients and consumers want to take part. We have experience with online and media-based recruitment of study patients. We will collaborate with international colleagues (eg, the United Kingdom). In a later phase, we can also recruit through our self-assessment app (VB1). Another threat will be the sensitive and private nature of the topic of pornography consumption and the reliability of the participants’ responses. We will build a reliability check in the questionnaire. This WP needs extensive sexological work. This will be possible by recruiting a PhD student with a sexology background and 2 extra sexologists purely for the assessment of our patients.

#### WP4: Risk Analysis and Contingency

First, there is (in theory) a risk that the scale development and validation will not succeed. However, the involved supervisors have extensive experience with developing or testing internationally published scales [85,86]. Second, WP4 relies heavily on school collaboration. The team has extensive experience with sensitive school-based research (eg, sexting), and has a large collaboration network of schools. Third, discomfort while participating may be experienced. The team is experienced in conducting safe and ethical research and to provide help in case of discomfort.

Looking ahead, the SWYPPe consortium will ensure that findings are actively disseminated across academic, clinical, educational, and societal domains. Beyond scientific publications and conference presentations, results will be translated into evidence-based tools, digital applications, and educational materials that can be used by adolescents, parents, teachers, and health care professionals. Importantly, the project’s data management plan was developed in the spirit of open and responsible data use—upon project completion, other researchers will be able to submit proposals to access and further analyze the SWYPPe data within a predefined ethical and governance framework. This ensures that any future research builds on the same neutral, interdisciplinary, and value-sensitive principles that guide this study. Through these dissemination and data-sharing efforts, the project aims to foster informed pornography literacy, promote sexual well-being among young people in Flanders and beyond, and contribute to a more balanced international dialogue on pornography and sexual development.

### Valorization Goals

A key ambition of the SWYPPe project lies in its strong emphasis on valorization, ensuring that the scientific knowledge generated across the 4 WPs is actively translated into concrete societal impact. Given the complexity and sensitivity of pornography consumption and its potential effects on sexual well-being among adolescents and young adults, valorization efforts will target a wide range of end users—youth themselves, educators, parents, health care professionals, and policymakers. From the onset, SWYPPe was developed as a multistakeholder project grounded in real-life concerns and voiced needs, and its valorization strategy aims to return the knowledge produced to the communities and sectors it seeks to inform and support.

The overall societal valorization goals of SWYPPe are threefold. First, we aim to normalize the discussion around pornography in both public and professional domains by generating balanced, evidence-based insights into its place within the sexual development and well-being of adolescents and young adults. This includes countering polarized narratives that either pathologize or trivialize pornography consumption, and instead fostering nuanced understandings of the diversity of pornography consumption patterns and experiences. Second, we seek to increase media and pornography literacy across the educational, parental, and health care landscape. This includes equipping adolescents and young adults with critical tools to self-reflect on their consumption and experiences, and enabling key adults in their lives to more effectively support this process. Third, we aim to improve the quality of care for adolescents and young adults presenting with sexual concerns that may be related to (or confounded by) pornography use, by contributing to clinical tools and training that support context-sensitive assessment and guidance.

To achieve these goals, SWYPPe will deliver a range of concrete outputs tailored to different professional and community needs. These include the following.

First, an interdisciplinary, evidence-based porn literacy lesson plan, designed for secondary education and cocreated with students and teachers from diverse sociocultural backgrounds. The lesson plan will be inclusive, age-appropriate, and sensitive to varying norms and experiences. It will integrate insights from WPs 1, 2, and 4, with particular attention to navigating the potential benefits and harms of pornography consumption, promoting critical engagement, and supporting sexual agency and respect.

Second, a self-assessment tool for pornography consumers, aimed primarily at adolescents and young adults, to allow for reflection on consumption patterns, motivations, and experienced impact. This digital tool, grounded in insights from WPs 1 and 3, will help distinguish between problematic, functional, and neutral pornography consumption. It will be designed in a user-friendly, nonjudgmental format and will provide tailored feedback and guidance, including when and where to seek professional help.

Third, a family communication tool to support open and respectful parent-child dialogue around pornography. Developed through cocreation (WP2), this tool will address known barriers to intrafamily communication and incorporate empirically grounded strategies for trust-based conversations, aiming to serve as both an educational and relational bridge.

Fourth, training materials and decision-making tools for health care providers, focused on assessing and managing sexual difficulties that may be associated with pornography consumption. These include a screening tool for clinicians (WP3), a clinical diary protocol for assessing patterns of arousal and function, and a set of psychoeducational modules. These resources will support professionals in avoiding reductive or moralizing frameworks and instead foster context-sensitive, evidence-based care.

Fifth, a validated Pornography Literacy Scale that can be used in future research and educational settings to measure the level of critical engagement and understanding among adolescents and young adults. This tool will facilitate longitudinal evaluation of educational initiatives and public health interventions.

Sixth, dissemination activities and public campaigns, including a youth-centered awareness campaign developed in collaboration with key stakeholders (eg, Sensoa and Pimento), to promote informed and responsible pornography use and highlight support options for those who struggle with their pornography consumption. Public-facing materials (infographics, short videos, social media content) will reflect the diversity and lived realities of Flemish youth.

SWYPPe’s valorization strategy is guided by a theory of change approach, linking all outputs to intended short-, medium-, and long-term outcomes, such as improved sexual well-being among adolescents and young adults, reduced stigma around pornography consumption–related concerns, and increased professional capacity across education and health care systems. The valorization team includes a dedicated valorization manager and representatives from each WP, ensuring continuous coordination, feedback, and stakeholder involvement throughout the project life cycle. All tools will be iteratively developed and tested through user-centered design methodologies, with attention to accessibility, inclusivity, and scalability.

By embedding valorization into the core of the project, SWYPPe not only seeks to generate new knowledge but to actively shape a more supportive, informed, and inclusive environment for young people navigating sexuality in the digital age.

## Supplementary material

10.2196/80058 Multimedia Appendix 1 Further explanation of work packages.

10.2196/80058 Checklist 1 PRISMA 2020 checklist with regards to work package 4. RO 4.1 Scoping Review on Porn Literacy Programs.

10.2196/80058 Checklist 2 SPIRIT 2025 checklist of items to address in a randomized trial protocol work package 3 RO 3.2.2 Understand the possible role of porn.

## Abbreviations

BVU: Belgische Vereniging voor Urologie
COREQ: Consolidated Criteria for Reporting Qualitative Research
DSM-5: Diagnostic and Statistical Manual of Mental Disorders, Fifth Edition
ERIC: Education Resources Information Center
FAIR: Findable, Accessible, Interoperable, and Reusable
HCP: health care practitioner
ICD-11: International Classification of Diseases, 11th Revision
IDI: in-depth interview
KULeuven: Katholieke Universiteit Leuven
PRISMA-P: Preferred Reporting Items for Systematic Reviews and Meta-Analysis Protocols
PRISMA-ScR: Preferred Reporting Items for Systematic Reviews and Meta-Analyses extension for Scoping Reviews
PXL: Hogeschool PXL
RCT: randomized controlled trial
REDCap: Research Electronic Data Capture
RO: research objective
SID: scientific input and discussion
SPIRIT: Standard Protocol Items: Recommendations for Interventional Trials
STROBE: Strengthening the Reporting of Observational Studies in Epidemiology
SWYPPe: sexual well-being of young people in times of widespread porn use
UAntwerpen: University of Antwerp
UGent: Ghent University
VVOG: Vlaamse Vereniging voor Obstetrie en Gynaecologie
VVS: Vlaamse Vereniging voor Seksuologie
WP: work package
