# Supplementary material for: Sexual Well-Being of Young People in Times of Widespread Pornography Use: Protocol for a Multidisciplinary Research Framework
Source: JMIR Res Protoc. 2026 May 4;15:e80058. doi: 10.2196/80058 (PMC13138793; doi:10.2196/80058)
Supplement: Multimedia Appendix 1 [file resprot-v15-e80058-s001.docx]

| RO | Research objectives | RQ | Research questions |
| --- | --- | --- | --- |
| **1.1.** | To assess the incidence of pornography consumption in society on an individual as well as on a sub-group level | 1A  1B | To what extent, and how and why is porn consumed within various (majority and minority) populations?  Can we identify pornography consumption patterns/sexual outcomes, based on gender, ethnicity, sexuality (sexual orientation/preferences), age, disability and class? |
| **1.2.** | To explore the associations between pornography consumption, self-perceived sexual functioning, and well-being | 1C  1D | What are risk as well as protective factors associated with self-perceived sexual functioning? In which way is self-perceived sexual functioning linked to pornography?  Under which conditions and how is pornography consumption associated with well-being (e.g. personal growth, positive relations, self-acceptance, relationship satisfaction)? |
| **1.3.** | To understand, from an intersectional perspective, how pornographic material is perceived and experienced among minoritized groups. | 1E  1F | How do social categories (gender, sexuality, age, ethnicity) interact/affect the way in which porn is read, used, accepted or disapproved of?  How do people with diverse religious/cultural backgrounds experience pornography? What is perceived as problematic and not? How do adolescents and young adults in minoritized groups negotiate between different expectations and norms? |
| **1.4.** | To explore how pornographic representations of minoritized groups affect their experiences of personal well-being | 1G | To what extent/or under which conditions do gender, sexual (e.g., sexual orientation and -preferences) and racialized minorities experience porn as objectifying and/or empowering? |

| RO | Research objectives | RQ | Research questions |
| --- | --- | --- | --- |
| **2.1.** | Map available evidence on the impact of online pornography use on sexual development and wellbeing of adolescents and young adults and identify best practices to deal with its potential (adverse) effects | 2A | What is the scientific evidence on the impact of online pornography use on the sexual development and wellbeing of adolescents and young adults? |
|  |  | 2B | What good practices are available to deal with adverse effects of online pornography use among young people? |
| **2.2.** | Examine the possible effects of online pornography on the (sexual) development of young people | 2C | What are the effects of online pornography consumption on sexual development and sexual wellbeing, including sexual self-image and perception on sexuality and relationships, according to adolescents and young adults? |
|  |  | 2D | What are key obstacles and good practices that young people encounter when discussing pornography with their parents? |
|  |  | 2E | What are preferred interfamily strategies to counter potential adverse effects of online pornography use according to young people? |
| **2.3.** | Examine the needs of parents in dealing with online pornography use of their children | 2F | What are parents’ current mediation strategies when it comes to their children’s online pornography consumption and what strategies do they prefer to enhance sexual development and sexual wellbeing of their children? |
|  |  | 2G | What are the parents’ needs and preferences in educating their children about online pornography and what do they perceive as appropriate? |
| **2.4.** | Inform the development of a tool to enhance parent-child communication on online pornography consumption | 2H | Does the proposed tool fit the needs of parents and does it help parents and children communicate with each other on online pornography? |

| RO | Research objectives | RQ | Research questions |
| --- | --- | --- | --- |
| **3.1.** | To assess the **prevalence** of pornography consumption in adolescents and young adults presenting with (not purely physical) sexual problems | 3A | What is the extent of (problematic) pornography consumption or abstinence in patients (and their partners) who present with sexual problems? Could a standardized assessment of pornography consumption benefit those patients? |
|  |  | 3B | Are different sexual problems associated with different patterns of and responses to pornography use and/or deliberate pornography abstinence (e.g., frequency, duration, solo/partnered, attitudes, expectations, masturbation combined pornography consumption or not, etc.)? |
| **3.2.** | **Understand** the possible role of pornography consumption in Aya's with situational physical arousal or orgasm problems. | 3C | What is the connection between (problematic) pornography consumption /porn abstinence and sexual response in Aya's with sexual problems related to arousal or orgasm? |
|  |  | 3D | Does pornography use/abstinence play a role in self-perceived sexual (dys)functions? |
|  |  | 3E | What are the links between pornography consumption and all changes in consumption pattern, including sudden total abstinence) and sexual problems and mental well-being? |
|  |  | 3F | To what extent do adolescents and young adults or their partners consider their pornography consumption problematic and/or contributing to their sexual problems? |
|  |  | 3G | Are adequate levels of physical/subjective arousal still attainable for Aya's with sexual problems connected to PUH, when engaging in real life sex with their partners of choice? |
|  |  | 3H | Are adequate levels of subjective arousal still attainable for adolescents and young adults with frequent pornography consumption (and problematic pornography consumption) when they refrain from pornography consumption during masturbation? |
|  |  | 3I | Can we differentiate between subgroups of patients in whom pornography consumption is positively or negatively affecting their sexual wellbeing and function or having no clinically relevant effect at all? |
| **3.3.** | **Develop** a screening tool, diary measure, and **psychophysiological procedure** to evaluate to role of porn use in case of physical arousal problems. | 3J | Are different psychophysiological sexual response patterns (e.g., high habituation proneness (physically and context generated), high sensitivity to distraction, and/or high need for novelty/specific stimuli) indicative of different subtypes of situational physical arousal problems? If so, how can care providers efficiently differentiate during a diagnostical process? |
|  |  | 3K | How do objectively assessed sexual response patterns and diary-based reports of sexual function and response at home relate to each other? |
|  |  | 3L | What is the relative contribution of a pornography consumption screening tool and psychophysiological arousal assessment to clinical decision making, in terms of assessment and diagnostics, in people of all genders presenting with physical arousal problems? |
|  |  | 3M | Can a screening tool be developed to help determine if changes in pornography consumption (e.g., frequency, content, context) might be desirable/possible/beneficial in (subsets of) patients with sexual problems? |
| **3.4.** | **Explore** normative thinking patterns of adolescents and young adults and healthcare providers about porn’s effects on sexual functioning. | 3N | Which factors may increase the probability that people consider themselves as being positively or negatively affected by pornography? |

| RO | Research Objectives | RQ | Research Questions |
| --- | --- | --- | --- |
| **4.1.** | Create an inventory and evaluation of existing pornography literacy education programs | 4A | Which educational programs about pornography literacy do already exist? |
|  |  | 4B | Which topics do current pornography literacy programs cover? Which are areas for future development? |
|  |  | 4C | What is the didactical and pedagogical quality of existing programs? Which evidence-based programs have been developed? Which improvements can future programs make? |
| **4.2.** | Develop and validate a measurement for porn literacy | 4D | How can we define and measure pornography literacy? |
| **4.3.** | Assess the diverse needs of adolescents and teachers regarding inclusive pornography literacy education | 4E | To what extent are teachers already implementing pornography literacy initiatives during sexual and relationship education? |
|  |  | 4F | Which challenges, needs and expectations regarding pornography literacy education do teachers perceive? |
|  |  | 4G | What information do adolescents and young adults expect during pornography literacy education? |
|  |  | 4H | What kind of pedagogical approaches do they prefer during pornography literacy education? |
| **4.4.** | Inform the development of an inclusive evidence-based lesson plan on porn literacy | 4I | Is the developed lesson plan effective in increasing porn literacy among adolescents and young adults? |
|  |  | 4J | Is the developed lesson plan effective in affecting pornography-related attitudes & behavioral intentions, pornography consumption behaviors? |
| **4.5.** | Inform the development of a tool that allows individuals to monitor their pornography consumption and make a self-assessment of the associated risk factors. | 4K | How do pornography consumers want to self-evaluate their pornography behavior? |
|  |  | 4L | How can an effective self-assessment tool on the positive and negative impact of pornography use be developed? |
